# What Is the Carbon Footprint of Adult Spinal Deformity Surgery?

**DOI:** 10.3390/jcm13133731

**Published:** 2024-06-26

**Authors:** Hiroyuki Nakarai, Cole Kwas, Eric Mai, Nishtha Singh, Bo Zhang, John C. Clohisy, Robert K. Merrill, Anthony Pajak, Jerry Du, Gregory S. Kazarian, Austin C. Kaidi, Justin T. Samuel, Sheeraz Qureshi, Matthew E. Cunningham, Francis C. Lovecchio, Han Jo Kim

**Affiliations:** 1 Department of Spine Surgery, Hospital for Special Surgery, New York, NY 10021, USA; 2 Department of Orthopaedic Surgery, The University of Tokyo, Bunkyo-ku, Tokyo 113-8655, Japan

**Keywords:** scoliosis, greenhouse gas, environmental impact, life cycle assessment, sustainability

## Abstract

**Background/Objectives**: While the economic cost of adult spinal deformity (ASD) surgery has been studied extensively, its environmental impact is unknown. The aim of this study is to determine the carbon footprint (CF) associated with ASD surgery. **Methods**: ASD patients who underwent > four levels of corrective surgery between 2017 and 2021 were included. The open group included a posterior-only, single-stage technique, while the minimally invasive surgery (MIS) group was defined as the use of lateral interbody fusion and percutaneous posterior screw fixation. The two groups were propensity-score matched to adjust for baseline demographic, surgical, and radiographic characteristics. Data on all disposables and reusable instruments, anesthetic gas, and non-gas medications used during surgery were collected from medical records. The CF of transporting, using, and disposing of each product and the footprint of energy use in operating rooms were calculated. The CF produced was evaluated using the carbon dioxide equivalent (CO_2_e), which is relative to the amount of CO_2_ with an equivalent global warming potential. **Results**: Of the 175 eligible patients, 15 pairs (65 ± 9 years, 47% female) were properly matched and analyzed for all variables. The average CF generated per case was 147.7 ± 37.3 kg-CO_2_e, of which 54% was attributable to energy used to sterilize reusable instruments, followed by anesthetic gas released into the environment (17%) and operating room air conditioning (15%). **Conclusions**: The CF generated during ASD surgery should be reduced using a multidisciplinary approach, taking into account that different surgical procedures have different impacts on carbon emission sources.

## 1. Introduction

Studies have shown that greenhouse gas (GHG) emissions pose a growing threat to public health through their impact on climate change [1,2]. The healthcare industry comprises a significant proportion of GHG emissions globally both directly from healthcare facility operations and indirectly from the supply chain of healthcare materials and services [3]. The health sector of the US specifically contributes the greatest GHG emissions of any individual country’s healthcare industry, comprising 25% of health sector emissions globally [4]. This has been trending upward over the past decades, with the US healthcare industry’s GHG emissions increasing at a faster rate compared to that of any other industrialized country, with an estimated 6% growth between 2010 and 2018 [5]. As such, there is significant interest in reducing contributions of GHG emissions from the healthcare industry in order to reduce costs and improve overall health. To achieve this, having a firm understanding of the carbon footprint (CF) of specific processes, such as adult spinal deformity (ASD) surgery, is essential.

While the economic cost of ASD surgery has been studied extensively, the environmental cost is unknown [6,7,8,9]. Although the environmental impact of healthcare services is multifaceted, Moldovan et al. developed a novel comprehensive framework for assessing the environmental sustainability of healthcare services [10]. They divided the factors for environmental sustainability into eight domains. Studying the CF generated in the OR would be included in the assessment of RA4 (mechanisms for monitoring energy consumption and waste generation). The goal of sustainability in this domain is achieved by reducing carbon emissions, the amount of waste in landfills, and energy usage. Since ASD surgery appears to be among the most environmentally impactful spine surgeries due to the use of the largest amount of medical supplies, implants, anesthetics, and energy consumption in the operating room (OR), it is valuable to estimate the amount of CF produced during ASD surgery [7,8,11,12]. Previous studies have evaluated the environmental impact of surgical procedures within the different disciplines of orthopedics including joints, trauma, and hands [13,14,15,16]. Based on the current literature, few studies have assessed the CF of spine surgery. For example, Wang et al. retrospectively studied the CFs in single-level transforaminal lumbar interbody fusions by evaluating carbon dioxide equivalents from anesthetic agents used during cases [17]. While the type and amount of anesthesia are important contributors to the environmental impact of spine surgery, there are other factors that must be accounted for in order to obtain a more comprehensive understanding of its true CF. To our knowledge, no other study has provided a comprehensive evaluation of the CF associated with ASD surgery through an assessment of the different phases of disposables, reusable instruments, and energy consumption in the OR. The purposes of the current study are to (1) determine the CF associated with ASD surgery and (2) compare the sources of CFs between minimally invasive surgery (MIS) and traditional open surgeries. We hypothesized that a greater amount of CFs would be generated in minimally invasive ASD surgery compared to open surgeries due to the greater number of reusable instruments used and the longer anesthesia time associated with the intraoperative positional change.

## 2. Materials and Methods

### 2.1. Patient Selection

This is a retrospective study using a registered patient database, and approval was obtained from the institutional review board of our hospital (IRB #2018-1142, approved on 1 August 2018). ASD patients who underwent > 4 levels of corrective surgery between 2017 and 2021 were included. Those with prior lumbar fusion > 2 levels were excluded. The open group included a posterior-only, single-stage technique, while the MIS group was defined as the use of lateral interbody fusion (LIF) and percutaneous posterior screw fixation.

### 2.2. Carbon Footprint Calculations

**Figure 1:**
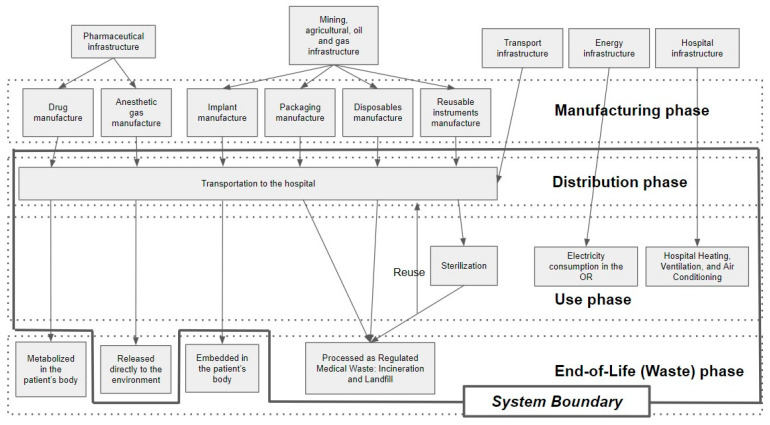
The system boundary defined the inclusions and exclusions for life cycle assessment (black line). Dotted line shows four phases of carbon emissions derived from each product.

We defined the functional unit of our study as ASD surgery performed in a hospital in New York, United States, based on the International Organization for Standardization (ISO, Geneva, Switzerland)-14040 standard [18], which is widely used as the standard protocol for life cycle assessment (LCA) [19]. Although all processes associated with the product should ideally be included, the data and cost constraints make this infeasible. The CFs produced from the distribution, use, and end-of-life (waste) phases of each product were included in the analyses (Figure 1). In our study, it was not possible to calculate the CF generated during the manufacturing phase of each product due to the lack of available information. The CF generated from the disposal of surgical implants, bone-graft products, and non-gas intravenous medications was excluded since they are implanted or metabolized in the patient’s body after surgery. Thus, the system boundary that defines the inclusions and exclusions for LCA was determined (Figure 1). Consequently, the following CFs were calculated: (1) single-use disposable items and reusable instruments from distribution to the end-of-life phase; (2) non-gas medications from distribution to the use phase; (3) anesthetic gas from distribution to the end-of-life phase; and (4) energy consumption in the OR during the use phase. The CFs were evaluated using the carbon dioxide equivalent (CO_2_e), which is relative to the amount of CO_2_ with an equivalent global warming potential. To calculate the CFs, Umberto ver. 11.9.2 (iPoint, Reutlingen, Germany) LCA modeling software and the Ecoinvent database ver. 3.9.1 (ecoinvent, Zurich, Switzerland) were used.

### 2.3. Single-Use Disposable Items, Reusable Instruments, and Non-Gas Medications

All disposable items, surgical implants, reusable instruments, and non-gas medications used were obtained from medical records. The disposable items included disinfection kits, surgical drapes, patient-warming devices, anesthesia kits, suction tubes, cell-salvage instruments, syringes, catheters, neuromonitoring electrodes, skin markers, monopolar/bipolar cord, navigation devices, sponge gauze, burr drill bits, surgical knives, surgical gloves and gowns, guide wires, pins, single-use instrumentation kits, hemostats, drain tubes, sutures, staplers, and wound dressings. Surgical implants included pedicle screws, set screws, rods, cages, connectors, and bone-grafting products. Any medications and colloid/crystalloid fluids used during the procedures were also collected. The weight of each item and its packaging were measured. To simplify the model, it was assumed that products made in the US or Canada were transported by trucks, while products manufactured outside the US or Canada were transported by freight container ships (see Appendix A). For each product, Google Maps (Alphabet Inc., Mountain View, CA, USA) was used to calculate the distance between the manufacturing site and our institution to assess the CF generated to distribute the products. The CF associated with transporting loaned instruments to and from our institution was also accounted for (see Appendix B) [20].

Our institution used steam sterilizers, and the amount of electricity consumed was calculated as electricity consumption (kWh) = 15.7 + 0.14 × mass (kg), as previously reported [21], and then converted to CO_2_e using the Ecoinvent database. Additionally, we accounted for the mass of the sterile drape, averaging 1.4 kg, for large instrument sets in a container [16]. 

Medical waste treatment practices differ among states and countries. New York State defines Regulated Medical Waste (RMW) as infectious human tissue waste, human blood and blood products, needles and syringes (sharps), cultures, stocks, or other infectious waste [22]. Based on the available data regarding the CF generated in permitted facilities, it was estimated that 1 kg of RMW emits 1.1 kg of CO_2_e during the waste process [22,23]. All disposables in ORs were treated as RMW in our institution. The reusable instruments were modeled to have a life cycle of 300 uses [20], and the footprint generated from the disposal of reusable instruments was divided by their life cycle to calculate the CF per case.

### 2.4. Inhalational Anesthetic Gas

Inhalational gas anesthetics are minimally metabolized and their waste products are vented directly to the environment, so the amount of gas used is approximately equal to the amount of gas released to the environment [17,19,24]. The amounts of anesthetic gas used were calculated from the electronic medical records and converted to CO_2_e using the following equation [17,25]:

CO_2_e = GWP_100_ × Time (min) × Free gas flow (L/min) × end-tidal gas concentration (%) × molar mass (g/mol) ÷ [2412 × Density (g/mL)]

where the 100-year global warming potential (GWP100) value indicates the effectiveness of each gas in capturing heat in the Earth’s atmosphere over a century, serving as the conversion unit for each agent compared to CO_2_ (see Appendix C). The CF from using oxygen gas and compressed air for anesthesia was also calculated based on the energy requirements for liquid oxygen (0.001 kWh/L) and compressed medical air (0.0003 kWh/L) [19].

### 2.5. Energy Consumption in ORs

Our institution’s HVAC system’s energy consumption was estimated to be 0.25 kWh/h/m^2^, based on the average energy consumption in ORs at two North American institutions [26,27]. The CFs related to lighting (0.329 kWh/h), patient-air warmers (0.8 kWh/h), and anesthesia machines (0.08 kWh/h) were also accounted for [19,28]. The power consumption for fluoroscopy (Ziehm vision RFD: Ziehm Imaging, Nuremberg, Germany) was calculated by multiplying the intraoperative fluoroscopy time by 25 kW, and the power consumption for the surgical robot (ExcelsiusGPS: Globus Medical, Audubon, PA, USA) was calculated by multiplying the operative time by 0.72 kW, based on the data provided in product brochures [29,30].

### 2.6. Statistical Analyses

When patients underwent staged surgery, the CFs of the two surgeries were combined. Student *t*-tests or Welch’s *t*-test were used to compare continuous variables between the two groups. Categorical variables were compared using Fisher’s exact tests. Since open surgery is usually indicated for more severe deformities and requires longer instrumentation than MIS surgery, the two cohorts were propensity-score matched based on 8 variables, including age, sex, body mass index (BMI), the upper instrumented vertebral level (UIV), the number of fused segments, preoperative pelvic incidence (PI), PI-LL mismatch, and the maximum coronal Cobb angle. A linear regression model was used to estimate the effect of increased anesthesia time on a time-dependent CF. To define a time-dependent CF, we excluded the CF associated with surgical implants, disposable items, reusable instruments, the use of fluoroscopic equipment, and the use of surgical robots, as these were not expected to increase with time. Instead, we included the CF associated with inhalational anesthetic gas, oxygen gas, medical compressed air, medications, OR electricity, and the HVAC system. Statistical analysis was performed using SAS ver. 9.4 (SAS, Inc., Cary, NC, USA). *p*-values of <0.05 were considered significant.

## 3. Results

### 3.1. Patient Characteristics and Surgical Factors

Of the 175 consecutive ASD patients (160 for the open group), 15 pairs (65 ± 9 years, 46.7% female) were properly matched (Table 1). In the MIS group, intraoperative repositioning between prone and lateral decubitus positions was carried out in seven (47%) patients, staged surgery in five (33%) patients, intraoperative navigation in thirteen (87%) patients, and robotic surgery in one (7%) patient, while none of the above was performed in the open group. The total operative time was significantly longer in the MIS group, while the estimated blood loss was significantly lower (Table 1). The anesthesia time was also significantly longer in the MIS group (474.0 ± 114.6 vs. 307.5 ± 57.3 min, *p* < 0. 001). The patients who underwent staged MIS surgery were found to have the longest median anesthesia time (*N* = 5, median 481 min, interquartile range (IQR) = 40), followed by those who required intraoperative repositioning (*N* = 8, median 416 min, IQR = 86) and single-position MIS surgery (*N* = 3, median 411 min, IQR = 93).

### 3.2. The CF Produced in ASD Surgery

**Figure 2:**
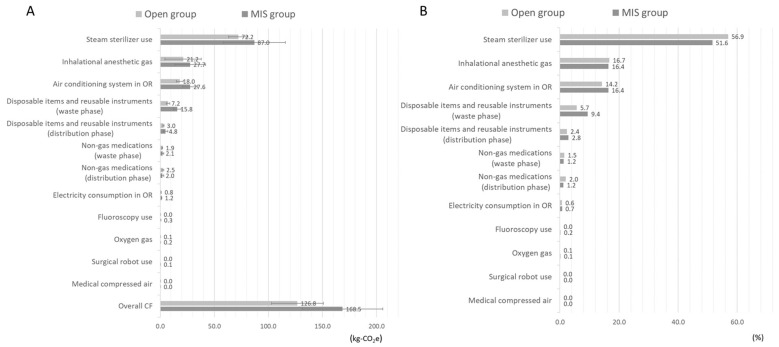
(**A**) The average amount of carbon footprint (CF) produced from multiple sources in the open and MIS groups is summarized in bars of standard deviations; (**B**) The percentage of CFs generated from each source in the open and MIS groups.

On average, each case utilized 62 disposable items, 38 reusable instrument packs/containers, 35 surgical implants, including bone-grafting products, and 46 non-gas medications. The MIS group consumed significantly more disposable and reusable items (Table 2). The average total amount of CF generated per case was 147.7 ± 37.3 kg-CO_2_e. The primary source of the CF production was the energy used to sterilize reusable instruments (54%), followed by anesthetic gasses released into the environment (17%), and the HVAC system in the OR (15%). The CFs resulting from product distribution, electricity consumption in the OR, including C-arm use and the HVAC system, and waste disposal processes were significantly higher in the MIS group compared to the open group, resulting in a significantly higher total generated CF (168.5 ± 37.2 vs. 126.8 ± 23.9 kg-CO_2_e, *p* = 0.001) (Figure 2A). Post hoc power analysis showed a power (1 − β) of 95.5% at a significance level of 0.05. The MIS group had a higher percentage of CFs related to the disposal process for disposable and reusable instruments compared to the open group (9.4% vs. 5.7%) (Figure 2B). Conversely, the open group had a higher percentage of CFs related to steam sterilizers (56.9% vs. 51.6%).

### 3.3. The Impact of Anesthesia Time on CF

**Figure 3:**
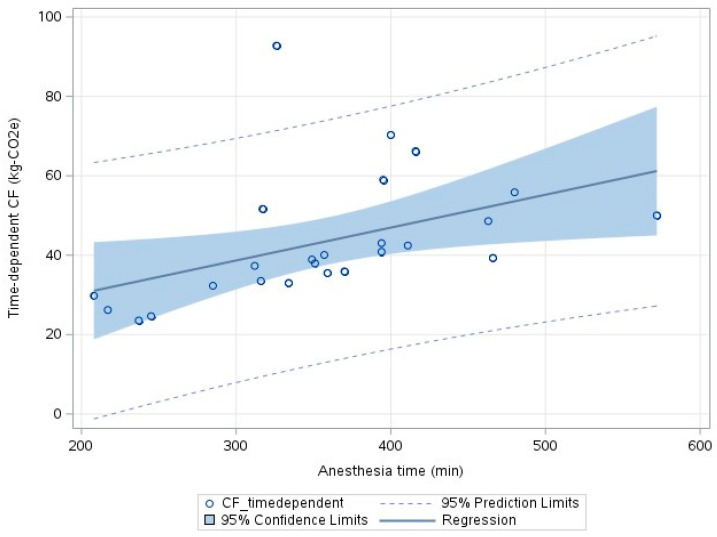
Time-dependent CFs, including CF related to inhalational anesthetic gas, oxygen gas, medical compressed air, medications, electricity, and air conditioning in the OR, are plotted versus anesthesia time. The regression line is within 95% confidence limits.

A linear regression model was used to assess the relationship between anesthesia time and a time-dependent CF in 25 patients. To prevent overestimation of the CF due to increased requirements of inhaled anesthetic gas and medications for anesthesia induction and extubation, five patients who underwent staged surgery were excluded. Linear regression modeling enabled the identification of time-dependent CFs that corresponded to anesthesia time based on the following equation:

Time-dependent CF (kg-CO_2_e) = 13.8 + 0.0828 × anesthesia time (min),

as shown in Figure 3 (r^2^ = 0.203).

## 4. Discussion

In our study, 15 pairs of ASD patients were included, and the CF of ASD surgery was estimated. Energy consumption for the sterilization of reusable instruments accounted for approximately 54% of the CF production, and the emission of anesthetic gases to the environment and the energy consumption of the HVAC system were also major contributors. The MIS group had a higher percentage of CFs related to the disposal process for disposable items and reusable instruments compared to the open group due to the larger number of items used, while the open group had a higher percentage of CFs related to steam sterilizers for reusable instruments. The linear regression model shows that extending the anesthesia time by one hour would result in an increase of 8.3 kg-CO_2_e produced in the OR.

The environmental impact of the healthcare sector encompasses a myriad of factors. However, surgery is known to be one of the most resource-intensive healthcare activities [3,15,26,31]. MacNeill et al. estimated the total CF generated in ORs in the US, UK, and Canada to be 9.7 million metric tons of CO_2_e per year [26]. Although the CFs of procedures have not been rigorously quantified, several studies have reported CFs related to surgical procedures. The annual CF of dermatologic surgery for skin cancer in Australia was estimated to be 8641 tons of CO_2_e [32]. The CF of three types of plastic surgery was estimated to range from 16.23 to 23.68 kg-CO_2_e [33]. Regarding the environmental costs of spine surgery, Talibi et al. found that an average of 8.91 kg of waste per case, equivalent to 24.5 kg-CO_2_e, was generated by neurosurgical procedures in England [11]. Notably, spinal fixation generated the largest amount of waste among all the neurosurgical procedures analyzed [11]. According to a previous study using the National Inpatient Sample database, 14,615 long-segment spinal fusion procedures involving ≥ nine vertebrae were performed in the US in 2014, a 141% increase from 6072 in 2004 [34]. Given that our study first clarifies that approximately 145 kg-CO_2_e is generated during ASD surgery, it was estimated that long-segment spinal fusion surgery, including ASD surgery, generates over 2000 metric tons of CO_2_e annually in the US, equivalent to approximately 238,000 gallons of gasoline consumed or 2,370,000 pounds of coal burned [35].

One of the main contributors to the CF in ASD surgery is the high use of disposable items and reusable instruments. According to Woods et al., the total CF of a robotic-assisted laparoscopy was 40.3 kg-CO_2_e, representing a 77% increase over conventional laparotomy, and the surgical robot consumed 20.30 kWh/h [36]. Similarly, MIS-ASD surgery appears to employ a greater number of instruments and disposable items compared to conventional open surgery, resulting in a greater CF. Leiden et al. compared the CF generated by single-use and reusable surgical instruments set for spinal fusion surgery [20]. They showed that the energy demand for steam sterilization of the reusable product had the highest impact on the CF, which is consistent with our study. According to McGain et al., optimizing the idle time of steam sterilizers effectively saved 26% of electricity usage, resulting in a reduction of 79 tons of CO_2_e emissions annually [21]. Additionally, only eight out of thirty-three instruments were utilized during dermatology surgeries, leaving 74% of the instruments unutilized [32]. Therefore, the optimization of steam sterilizer use and the minimization of unused instruments to reduce the number of reusable instruments sterilized may be effective in reducing the CF.

Due to the extended duration of ASD surgery, the CF of the anesthetic gas should be of concern. For a single-level transforaminal lumbar interbody fusion, the CF associated with general anesthesia was reported to average 4.73 kg-CO_2_e [17]. To mitigate the environmental impact of anesthetic gas, avoiding the use of N_2_O and unnecessarily high fresh gas flow rates has proven effective, as N_2_O is not only a heat-trapping gas but also an ozone-depleting agent [25]. In addition, the unnecessary use of desflurane should be avoided as it has a global warming potential approximately 20 times greater than sevoflurane [17]. Total intravenous anesthesia may provide benefits in decreasing the CF because the agents have a lower CF than inhaled gases [37]. Thiel et al. suggested that incorporating multiple approaches could help reduce the CF by reducing anesthetic gasses, maximizing instrument reuse or single-use device reprocessing, and reducing off-hour energy in the OR, which could reduce the CF of an average laparoscopic hysterectomy by up to 80% [38]. Hence, collaboration across specialties and professions is critical to reducing the CF and making ASD surgery more sustainable.

Several limitations should be noted. First, we excluded the CF generated during the manufacturing of the products in the current study. Previous studies that estimated the CF generated upstream in the supply chain based their calculations on material compositions [11,26,39]. However, the medical supplies utilized in spine surgery have become complex and consist of multiple materials and intermediate products. This makes it difficult to accurately estimate the CF during manufacturing, as the process is not usually publicly available. Therefore, further research is required to analyze the CF of manufacturing each product individually in order to avoid underestimating its environmental impact. Second, our results require cautious interpretation as the CF varies among states or countries due to the difference in the regulation of medical waste, costs of transportation, and the CF of electricity production [26]. Therefore, knowing that the CF may vary between institutions and countries is critical to devising the most efficient approach to reducing CFs, and future studies are needed to determine the CF associated with ASD surgery in different institutions and different countries to better understand the environmental impact.

## 5. Conclusions

For the first time, the CF generated during ASD surgery was estimated. The findings suggest that CF can be effectively reduced by optimizing the use of steam sterilizers and minimizing the number of extra reusable instruments, particularly in open surgery for ASD. In addition, reducing the number of disposable items and shortening the anesthesia time using the single-position technique may be effective in reducing CF, especially in MIS surgery. Reducing the CF is not the primary goal when compared to ensuring patient safety and economic efficiency; however, a multidisciplinary approach should begin to explore ways to eliminate unnecessary carbon emissions.

## Figures and Tables

**Table 1:** Patient characteristics before and after propensity score matching.

	MIS Group (N = 15)	Open Group before Matching (N = 160)	*p*-Value	Open Group after Matching (N = 15)	*p*-Value
Age, years, mean ± SD	66.0 ± 7.1	66.0 ± 9.9	0.99	64.5 ± 11.5	0.67
Sex, female, *n* (%)	7 (46.7)	113 (70.6)	0.08	7 (46.7)	1.00
BMI, kg/m	27.3 ± 4.8	27.3 ± 4.8	0.96	27.7 ± 4.5	0.82
UIV at lumbar spine, n (%)	14 (93.3)	26 (16.3)	<0.001	11 (73.3)	0.33
No. of fused segments	4.7 ± 0.6	7.8 ± 3.3	<0.001	4.9 ± 1.8	0.60
Preop PI, °	49.5 ± 10.9	55.0 ± 13.3	0.13	50.2 ± 13.6	0.89
Preop PI-LL mismatch, °	25.7 ± 15.3	22.2 ± 18.6	0.47	23.0 ± 12.5	0.60
Preop coronal Cobb, °	29.0 ± 13.4	37.2 ± 21.6	0.046	26.5 ± 18.1	0.67
Preop PT, °	25.7 ± 5.6	26.9 ± 10.1	0.50	23.9 ± 7.5	0.46
Preop TK, °	26.8 ± 13.3	35.5 ± 18.7	0.10	30.2 ± 13.2	0.50
Preop TPA, °	25.9 ± 10.1	26.1 ± 11.9	0.95	25.9 ± 22.9	0.41
SPO levels, n	0	3.1 ± 2.1	<0.001	1.9 ± 0.4	<0.001
EBL, g	285.0 ± 240.5	979.9 ± 670.3	<0.001	823.3 ± 510.9	0.002
Operative time, min	383.5 ± 100.0	260.3 ± 52	<0.001	235.1 ± 51.7	<0.001
Postop LL correction, °	15.7 ± 12.7	17.4 ± 15.6	0.69	15.5 ± 12.0	0.97
Postop TK correction, °	7.1 ± 11.4	4.6 ± 17.9	0.64	5.0 ± 13.2	0.70
Postop TPA correction, °	10.3 ± 9.3	20.4 ± 19.9	0.005	16.2 ± 11.9	0.20

MIS indicates minimally invasive surgery; SD, standard deviation; BMI, body mass index; UIV, upper instrumented vertebra; PI, pelvic incidence; LL, lumbar lordosis; PT, pelvic tilt; TK, thoracic kyphosis; TPA, T1 pelvic angle; SPO, Smith Peterson osteotomy; EBL, estimated blood loss.

**Table 2:** Number of medical resources consumed or utilized in the surgeries.

	MIS Group (*N* = 15)	Open Group (*N* = 15)	*p* Value
No. of non-gas medication, mean ± SD	48.1 ± 23.2	43.0 ± 9.2	0.44
No. of disposable items consumed	88.7 ± 25.4	34.3 ± 7.3	<0.001
No. of surgical implants *	31.1 ± 7.6	39.5 ± 11.4	0.025
No. of reusable instruments	43.9 ± 13.4	31.5 ± 3.7	0.003
Single-packaged instrument	5.7 ± 2.3	4.4 ± 0.9	0.06
Middle-sized tray of instruments	19.0 ± 7.6	16.2 ± 2.6	0.18
Container of instruments	19.2 ± 5.6	10.9 ± 1.2	<0.001

* Surgical implants included pedicle screws, set screws, rods, cages, connectors, and bone-grafting products.

## Data Availability

The data from this study are available upon reasonable request.

## Appendix A

We assumed that products outside the US or Canada were shipped from the manufacturing facility by truck to the largest port in the country, then by ship to the Port of New York and New Jersey, and then by truck to our institution.

The Port of New York and New Jersey, comprising Port Newark–Elizabeth Marine Terminal (20.4 miles from our institution), Port Jersey Marine Terminal (17.0 miles), Howland Hook Marine Terminal (24.1 miles), and Red Hook Marine Terminal (10.0 miles), was assumed to be used for importing supplies for overseas transportation. We calculated the average distance of 17.9 miles between our institution and the four ports.

The distance in nautical miles (nm) from the Port of New York and New Jersey to the primary exporting port in each country/region is as follows:

- North America
  · Puerto Rico: The Port of San Juan (1805 nm);
  · Dominican Republic: The Port of Caucedo (1732 nm).
- Europe
  · Germany and Switzerland: The Port of Hamburg (4195 nm);
  · Denmark: The Port of Aarhus (3975 nm);
  · Italy: Porto di Gioia Tauro (4658 nm);
  · Sweden: The Port of Gothenburg (3859 nm);
  · Spain: The Port of Algecuras (3557 nm).
- Asia
  · China: Shanghai Port (13,903 nm);
  · Hong Kong: Hong Kong Port (13,402 nm);
  · Malaysia: Port Klang (11,017 nm);
  · Singapore: The Port of Singapore (11,247 nm);
  · India: Mumbai Port (9078 nm).

## Appendix B

The distance between our institution and the storage facility was estimated to be the average distance from three airports and four ports in the New York metropolitan area, assuming that these storage facilities are located near transportation hubs.

The average distance between our institution and the storage site of the loaner instrument set is 18.0 miles. This was calculated based on the following distances: 8.7 miles from LaGuardia Airport, 18.7 miles from John F. Kennedy International Airport, 26.8 miles from Newark International Airport, and the distance from The Port of New York and New Jersey.

## Appendix C

	**Molar Mass (g/mol)**	**Density (g/mL)**	**GWP100 ***
Sevoflurane †	200.1	1.22	130
Isoflurane †	184.5	1.50	510
Desflurane †	168	1.47	2540
N_2_O †	44	1.98	298
* GWP100 value indicates the effectiveness of each gas in capturing heat in the Earth’s atmosphere over a century, serving as the conversion unit for each agent compared to CO_2_. † [40].			
